# Impact of Timing of Minor Amputations After Revascularization on Patient Outcomes

**DOI:** 10.7759/cureus.73947

**Published:** 2024-11-18

**Authors:** Rema AlRashed, Faisal A Albogomi, Faisal A Almudaiheem, Talal A Almutairi, Khalid A Albassam, Fahad K Aljaber, Hussam A Alharbi

**Affiliations:** 1 General Surgery, Prince Sultan Military Medical City, Riyadh, SAU; 2 Vascular Surgery, Prince Sultan Military Medical City, Riyadh, SAU; 3 General Surgery/Breast and Endocrine Surgery, Al Kharj Armed Forces Hospital, Riyadh, SAU

**Keywords:** amputation, cli, diabetic foot, endovascular, open repair

## Abstract

Background: Vascular complications from diabetes contribute significantly to major and minor limb amputations. Diabetes is a major health burden in Saudi Arabia, with increased incidence in rural areas. The purpose of this study was to evaluate the timing of minor lower limb amputations after revascularization and their relative outcomes.

Methods: This was a retrospective study done in Prince Sultan Military Medical City, Riyadh, Saudi Arabia. Patients who underwent minor lower limb amputation after revascularization during 2018-2022 were included in the study.

Results: Of the 90 patients who were eligible for the study, 83 (94%) were diabetic, and 66 (73%) underwent revascularization. The timing between revascularization and amputation varied, with 34 (37.8%) amputations occurring on the same day as revascularization, 17 (18.9%) occurring within seven days of revascularization, and 39 (43.3%) occurring more than seven days post-revascularization. No significant differences in amputation (p=0.105) were observed based on the timing of amputation after revascularization. Diabetic patients showed significantly higher rates of wound infection (p=0.028) and longer healing times (p=0.000). Finally, diabetic patients were more likely to have healing times of more than 60 days (25.9%) compared to non-diabetic patients (20.0%).

Conclusion: Based on our results, the timing of endovascular or surgical repair did not affect patient outcomes. Moreover, diabetes was found to be a prognostic factor for poor wound healing and infection.

## Introduction

Vascular compromise and complications of diabetes are considered major steps leading to major and minor amputations. Diabetes mellitus, in particular, represents a major burden as a disorder in Saudi Arabia with rising incidence, including in rural areas [1-3]. Therefore, the incidence of its subsequent complications, including amputations, is relatively increasing. In a 2012 study, the estimated number of lower limb amputations in Saudi Arabia was 3970 patients annually [4]. Although no recent sufficient data are available to estimate the prevalence of lower limb amputations in Saudi Arabia, multiple studies in the literature report an increasing incidence in different cities of the kingdom [5,6]. Moreover, the impact of this condition is not limited to the disease itself but also extends to disability and its effect on quality of life, leading to the initiation of amputation rehabilitation programs [7]. This health burden is also encountered globally. For example, in Australia, approximately 4190 amputations are performed annually, with a relative cost of $875 million per year to the healthcare system [8].

Despite international efforts to recognize the importance of such an entity, many key factors play a role in controlling this disease, including diabetes mellitus and peripheral vascular disease, which constitute a poor prognosis when combined as compared to either one occurring alone [9]. Although there has been an increase in endovascular procedures to improve blood flow and wound healing in diabetic patients with underlying peripheral vascular disease, thus decreasing the need for amputation, there is a lack of standardization or guidelines regarding the optimal time to perform lower limb amputations. Multiple studies have advocated for different optimal times for lower limb amputation post-revascularization, including 30 days, 60 days, and one year [8,10-12]. As there is no consensus or agreement regarding the timing of minor lower limb amputations after revascularization, the purpose of our study was to evaluate the timing of minor lower limb amputations after revascularization and its relative outcome.

## Materials and methods

This was a retrospective cohort study carried out in Prince Sultan Military Medical City (PSMMC), Riyadh, Saudi Arabia, in 2023. Approval to conduct the study was obtained from the institutional review board of PSMMC Scientific Research Center (approval number E-2148, August 2023). The study population was recruited from PSMMC files of all patients who underwent minor lower limb amputation after revascularization. Inclusion criteria included adult patients (≥18 years of age) who underwent minor amputation from 2018 to 2022. Minor amputation was defined as rays or toe amputations. The patients were further categorized based on whether revascularization was done. Data on the characteristics of the sample were then collected, including age, gender, diabetes status, peripheral and/or cardiovascular disease, and smoking history. Patients who refused amputation or underwent other types of amputation were excluded from the study.

Data from 2018-2022 were collected from the PSMMC Vascular and Endovascular Surgery Data Registry using a convenient, non-probability sampling technique. The data were then coded, entered, and analyzed using IBM SPSS Statistics for Windows, Version 27 (Released 2020; IBM Corp., Armonk, New York, United States). The study took one year to complete.

## Results

The study population comprised 90 individuals with a mean age of 68 (±12) years, with 67 (74.4%) male and 23 (25.6%) female patients. The majority of the participants had hypertension (n=75; 83.3%), and 40 (44.4%) patients had dyslipidemia. Approximately 19 (21.1%) patients had end-stage renal disease, and 63 (70.0%) had cardiovascular disease. Eighty-five (94.4%) participants had diabetes. Smoking was reported in 20 (22.2%) patients. Previous revascularization procedures were documented in 24 (26.7%) cases, with a small percentage having undergone multiple revascularizations. Approximately one-third of the participants had a history of amputation (n=30; 33.3%), as seen in Table 1.

**Table 1:** Demographic and clinical characteristics of the study population. SD: standard deviation, ESRD: end-stage renal disease, CVD: cardiovascular disease.

		n/Mean	%/SD
Age		68	12
Gender	Male	67	74.4%
	Female	23	25.6%
Hypertension	No	15	16.7%
	Yes	75	83.3%
Dyslipidemia	No	50	55.6%
	Yes	40	44.4%
ESRD	No	71	78.9%
	Yes	19	21.1%
CVD	No	27	30.0%
	Yes	63	70.0%
Diabetes	No	5	5.6%
	Yes	85	94.4%
Smoker	No	70	77.8%
	Yes	20	22.2%
Previous revascularization	No	64	71.1%
	Yes	24	26.7%
	2 revascularizations	1	1.1%
	3 revascularizations	1	1.1%
Previous amputation	No	60	66.7%
	Yes	30	33.3%

Table 2 presents the characteristics of amputations within the studied population. Of the individuals included, 66 (73.3%) underwent current revascularization; specifically, 48 (53.3%) underwent endovascular interventions, 16 (17.8%) underwent open surgery, and two (2.2%) underwent both endovascular and open surgery. The distribution of amputations revealed that two (2.2%) patients did not undergo amputation, 47 (52.2%) underwent minor amputations, and 41 (45.6%) underwent major amputations.

**Table 2:** Current amputation characteristics of the study sample.

		n	%
Revascularization	No	24	26.7%
	Yes	66	73.3%
Type of intervention	No intervention	24	26.7%
	Endovascular	48	53.3%
	Open surgery	16	17.8%
	Endovascular and open surgery	2	2.2%
Amputation	No	2	2.2%
	Minor amputation	47	52.2%
	Major amputation	41	45.6%
Urgency of amputation	Emergency	5	5.6%
	Semi-elective	80	88.9%
	Elective	5	5.6%
Time between revascularization and amputation	Same-day	25	37.8%
	Within seven days	12	18.9%
	More than seven days	29	43.3%

**Figure 1:**
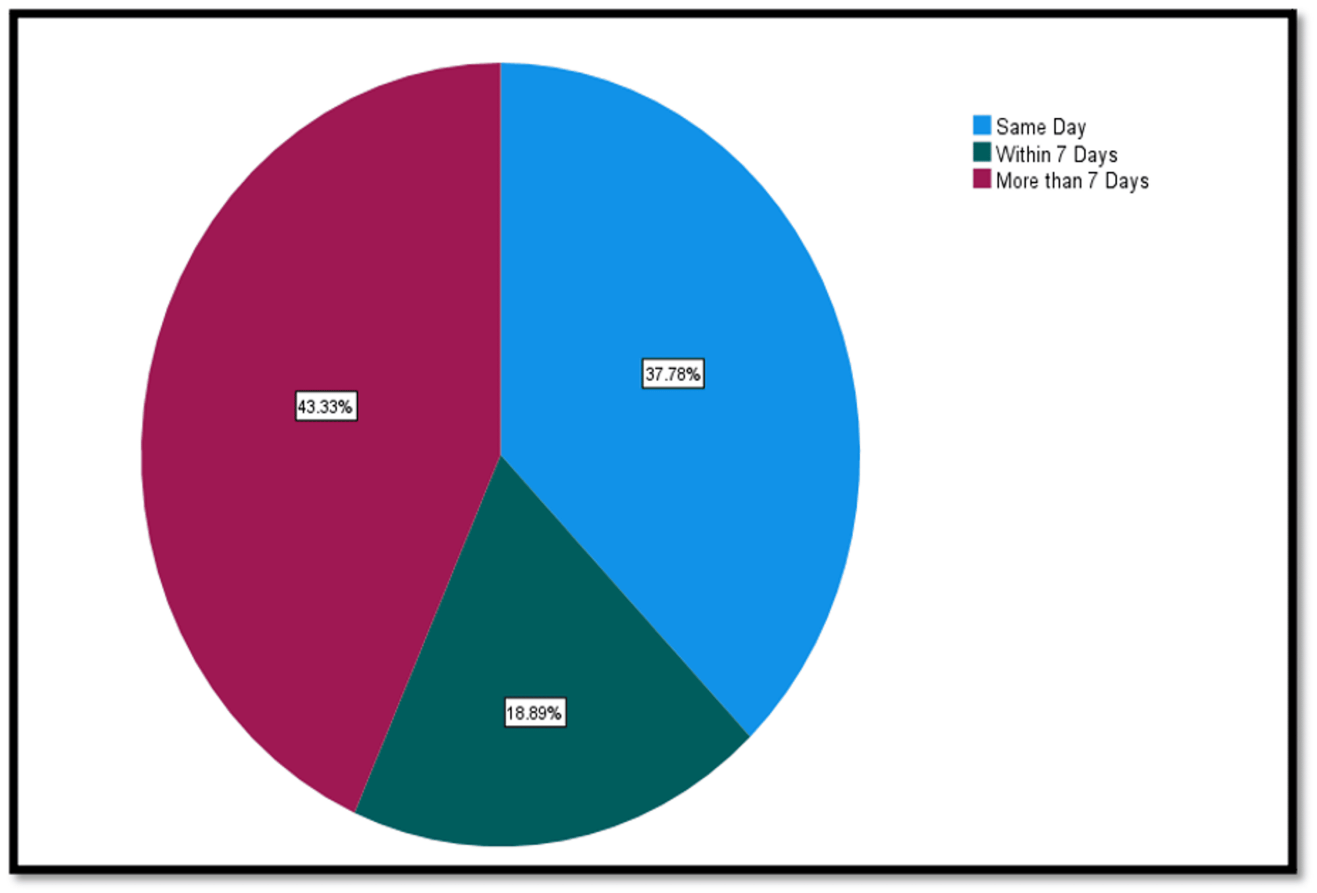
Time between revascularization and amputation.

Regarding the urgency of amputation, the majority of cases (n=80; 88.9%) were semi-elective, whereas five (5.6%) each were categorized as emergency or elective. The timing between revascularization and amputation varied, with 25 (37.8%) amputations occurring on the same day as revascularization, 12 (18.9%) occurring within seven days, and 29 (43.3%) occurring more than seven days post-revascularization, as seen in Figure 1.

**Figure 2:**
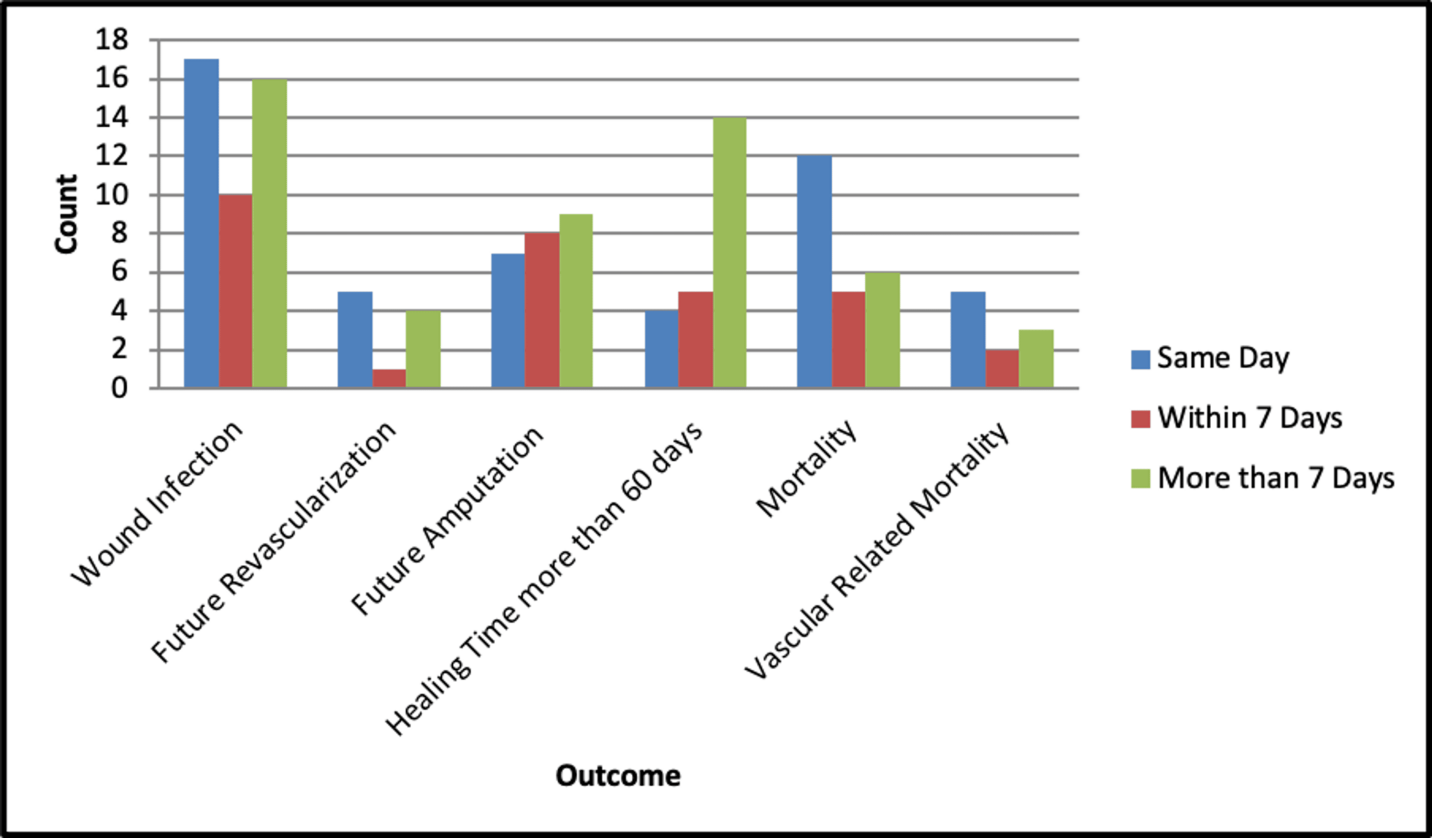
Stratification of outcomes based on time between amputation and revascularization.

Table 3 and Figure 2 present the clinical outcomes of amputation based on the timing between revascularization and amputation. Wound infection was observed in 17 (50.0%), 10 (58.8%), and 16 (41.0%) cases for amputations on the same day, within seven days, and more than seven days, respectively. No statistically significant differences were observed for the wound infection rate (p=0.447), future revascularization rate (p=0.624), future intervention type (p=0.686), or future amputation type (p=0.105) based on the timing of amputation after revascularization.

**Table 3:** Differences in clinical outcomes of amputation based on timing of amputation after revascularization. IQR: interquartile range.

		Time between revascularization and amputation						p-value
		Same Day		≤7 days		>7 days		
		n/Median	%/IQR	n/Median	%/IQR	n/Median	%/IQR	
Wound infection	No	17	50.0%	7	41.2%	23	59.0%	0.447
	Yes	17	50.0%	10	58.8%	16	41.0%	
Future revascularization	No	29	85.3%	16	94.1%	35	89.7%	0.624
	Yes	5	14.7%	1	5.9%	4	10.3%	
Type of future intervention	No intervention	29	85.3%	16	94.1%	36	92.3%	0.686
	Interventional radiology	1	2.9%	0	0.0%	1	2.6%	
	Endovascular	2	5.9%	0	0.0%	0	0.0%	
	Open surgery	2	5.9%	1	5.9%	2	5.1%	
Future amputation	No	27	79.4%	9	52.9%	30	76.9%	0.105
	Yes	7	20.6%	8	47.1%	9	23.1%	
Healing time	Unknown	25	73.5%	11	64.7%	19	48.7%	0.278
	≤30 days	1	2.9%	0	0.0%	2	5.1%	
	31–60 days	4	11.8%	1	5.9%	4	10.3%	
	>60 days	4	11.8%	5	29.4%	14	35.9%	
Mortality	No	22	64.7%	12	70.6%	33	84.6%	0.139
	Yes	12	35.3%	5	29.4%	6	15.4%	
Vascular-related mortality	No	29	85.3%	15	88.2%	36	92.3%	0.639
	Yes	5	14.7%	2	11.8%	3	7.7%	
Length of hospital stay		15	2(37–5)	21	17(31–14)	20	20 (29–9)	0.577

**Figure 3:**
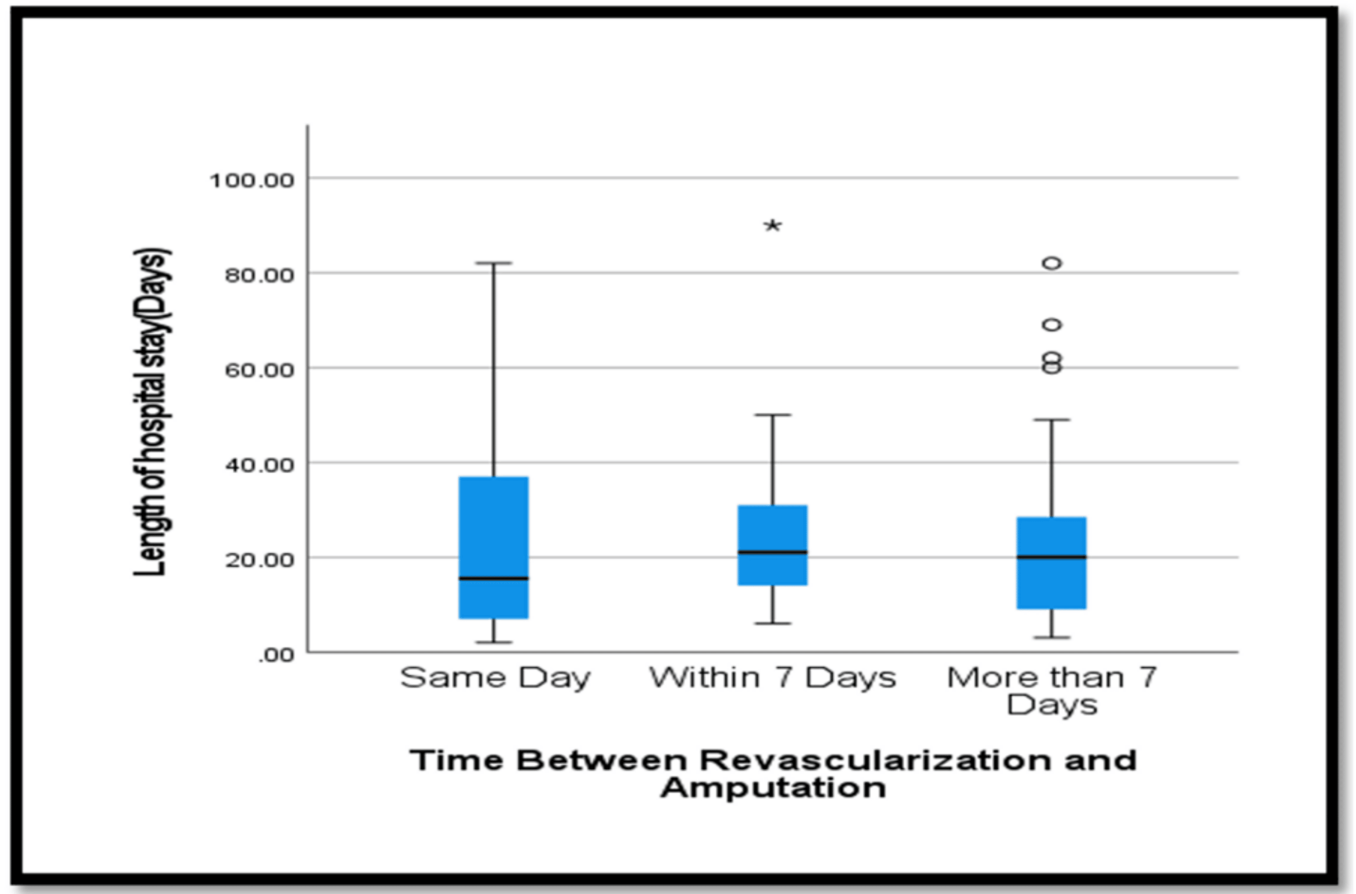
Length of hospital stay according to time between revascularization and amputation.

Healing time showed no statistically significant differences (p=0.278) across the three time categories. Mortality rates, both overall and vascular-related, tended to be higher in amputations performed on the same day post-revascularization, but these differences did not reach statistical significance (p=0.139 and p=0.639, respectively). No significant difference in the length of hospital stay was observed based on the time between revascularization and amputation (p=0.577), as seen in Figure 3.

**Figure 4:**
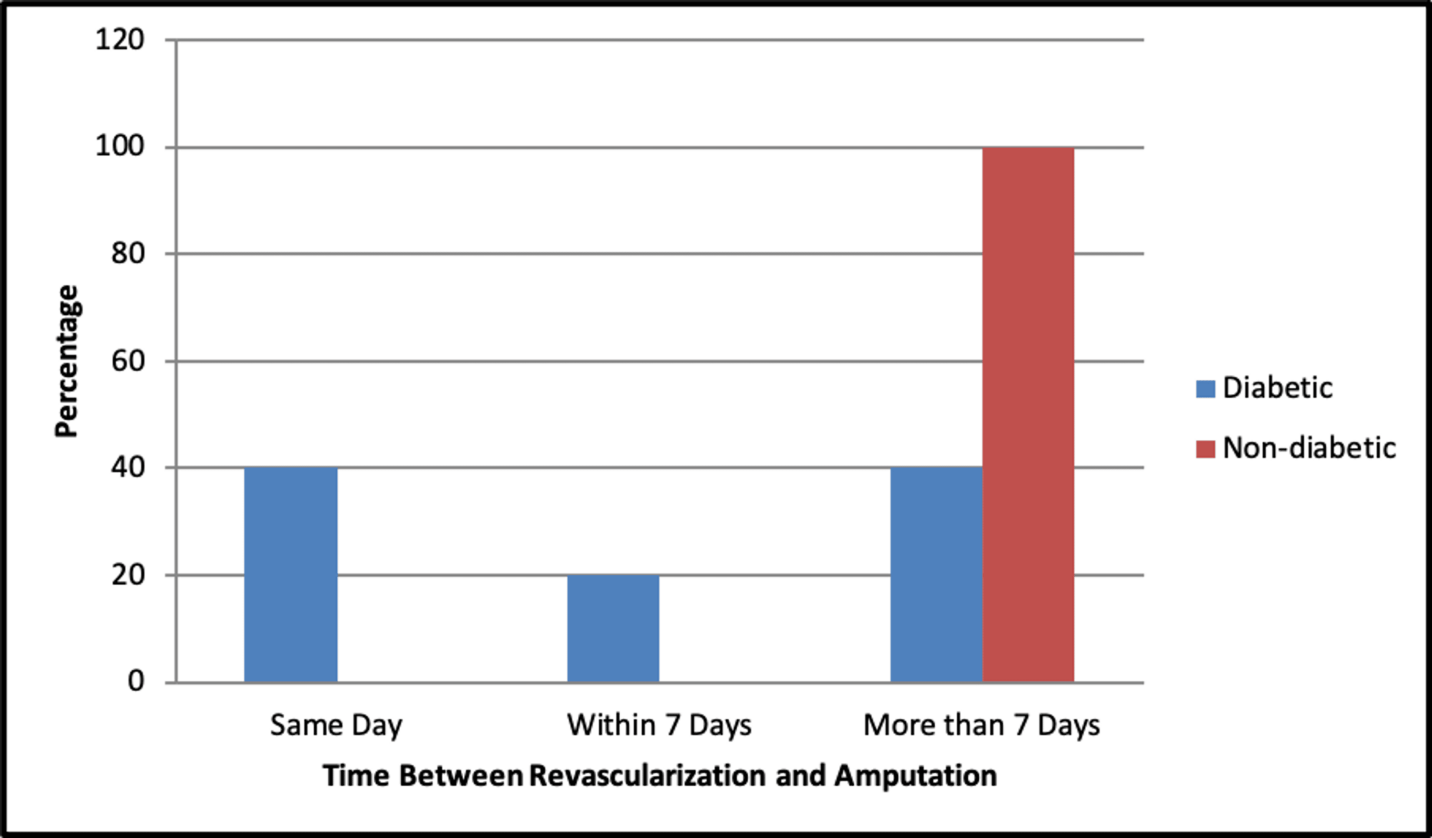
Comparison of time between revascularization and amputation between diabetic and non-diabetic groups.

Table 4 compares amputation and its outcomes between diabetic and non-diabetic individuals. The analysis revealed noteworthy differences between the two groups. Regarding current revascularization, there was no significant difference between diabetic and non-diabetic individuals (p=0.729). Similarly, there was no significant difference in the intervention type between the groups (p=0.906). However, a significant difference was observed in the occurrence of amputations between diabetic and non-diabetic individuals (p=0.021), where diabetic individuals had a higher rate of minor amputations (45.9%) compared to non-diabetics (40.0%). There was no significant difference in amputation urgency between the two groups (p=0.321). There was a significant difference in timing between revascularization and amputation between the diabetic and non-diabetic groups (p=0.032). Notably, 100% of non-diabetic individuals underwent amputation more than seven days post-revascularization, whereas 40.0% of diabetics had same-day amputations, and 40% had amputation seven days post-revascularization, as seen in Figure 4.

**Table 4:** Amputation and its outcomes among diabetic and non-diabetic patients.

		Diabetes				p-value
		No (N=5)		Yes (N=85)		
		n	%	n	%	
Current revascularization	No	1	20.0%	23	27.1%	.729
	Yes	4	80.0%	62	72.9%	
Intervention type	No intervention	1	20.0%	23	27.1%	.906
	Endovascular	3	60.0%	45	53%	
	Open surgery	1	20.0%	15	17.6%	
	Endovascular and open surgery	0	0.0%	2	2.4%	
Current amputation	No	1	20.0%	1	1.2%	.021*
	Minor amputation	2	40.0%	45	52.9%	
	Major amputation	2	40.0%	39	45.9%	
Urgency of amputation	Emergency	1	20.0%	4	4.7%	.321
	Semi-elective	4	80.0%	76	89.4%	
	Elective	0	0.0%	5	5.9%	
Time between revascularization and amputation	Same day	0	0.0%	34	40.0%	.032*
	≤7 days	0	0.0%	17	20.0%	
	>7 days	5	100.0%	34	40.0%	
Wound infection	No	5	100.0%	42	49.4%	.028*
	Yes	0	0.0%	43	50.6%	
Healing time	Unknown	2	40.0%	53	62.4%	.000*
	≤30 days	2	40.0%	1	1.2%	
	31–60 days	0	0.0%	9	10.6%	
	>60 days	1	20.0%	22	25.9%	
Mortality	No	4	80.0%	63	74.1%	.769
	Yes	1	20.0%	22	25.9%	

## Discussion

Historically, major amputations were the standard of care for lower limb gangrene, resulting in high morbidity and mortality rates. However, with advancements in minor amputations, outcomes have improved, especially when combined with revascularization [13].

In a study done by Chaing et al. [8], where revascularization was performed prior to minor amputations, revascularization done within 90 days of a minor amputation was associated with higher rates of limb preservation. In another study by Doyle et al., one-year limb survival reached 76.8% in the absence of infection and diabetes [10]. In our study, different time frames were used, as minor amputations were categorized as either on the same day of revascularization or ≤7 or >7 days following revascularization, which showed an increased incidence of future amputations across all time frames. However, this difference in future amputations was not statistically significant, which may be because the short time frames of the study did not affect wound healing or future further amputation.

It is well studied that diabetes is associated with impaired wound healing, ultimately leading to a high probability of wound infection [14]. Although non-diabetic patients represented a small proportion of our study population, healing time was not improved in diabetic patients even after revascularization, as observed in other studies [15,16]. In a study by Doyle et al., the optimal healing between revascularization and amputation was less than 30 days, and the authors emphasized that diabetes mellitus and end-stage renal disease were contributing factors for poor healing post-revascularization [10]. Elgzyri et al. also reported that revascularization resulted in significantly shorter healing times in cases of minor amputation within 60 days after revascularization in the absence of infection [12].

In a recent large-cohort study, the need for minor amputation was associated with a significant risk of future major amputation in the first year, and 50% mortality within five years [17], but this risk did not consider whether patients were revascularized. Furthermore, we found no statistical significance between revascularization and prevention of future amputations in our study, as reported in the literature [18].

Our study had some limitations. First, this was a small, single-center, retrospective cohort study, which may limit the generalizability of the results. Second, decisions regarding revascularization and the specific type of procedure chosen were made at the surgeon’s discretion, which is an inherently biased process. A longer follow-up is needed to further determine wound healing and the need for further intervention.

## Conclusions

Diabetes is a poor prognostic factor for wound healing and infection, regardless of revascularization, Based on our results, the timing of endovascular or surgical revascularization did not seem to change the patient’s outcome or mortality. Future large randomized controlled studies are necessary to mitigate potential bias and obtain more definitive evidence for the development of guidelines concerning the timing of revascularization.
